# Docking, MD Simulations, and DFT Calculations: Assessing W254’s Function and Sartan Binding in Furin

**DOI:** 10.3390/cimb46080486

**Published:** 2024-07-30

**Authors:** Nikitas Georgiou, Thomas Mavromoustakos, Demeter Tzeli

**Affiliations:** 1 Laboratory of Organic Chemistry, Department of Chemistry, National and Kapodistrian University of Athens, Panepistimioupolis Zografou, 11571 Athens, Greece; nikitage@chem.uoa.gr; 2 Laboratory of Physical Chemistry, Department of Chemistry, National and Kapodistrian University of Athens, Panepistimioupolis Zografou, 11571 Athens, Greece; 3 Theoretical and Physical Chemistry Institute, National Hellenic Research Foundation, 48 Vassileos Constantinou Ave., 11635 Athens, Greece

**Keywords:** furin, sartan, DFT, Induced-fit drug docking, molecular dynamics

## Abstract

Furins are serine *endoproteases* that are involved in many biological processes, where they play important roles in normal metabolism, in the activation of various pathogens, while they are a target for therapeutic intervention. Dichlorophenyl-pyridine “BOS” compounds are well known drugs that are used as inhibitors of human furin by an induced-fit mechanism, in which tryptophan W254 in the furin catalytic cleft acts as a molecular transition energy gate. The binding of “BOS” drug into the active center of furin has been computationally studied using the density functional theory (DFT) and ONIOM multiscaling methodologies. The binding enthalpies of the W254 with the furin-BOS is −32.8 kcal/mol (“open”) and −18.8 kcal/mol (“closed”), while the calculated torsion barrier was found at 30 kcal/mol. It is significantly smaller than the value of previous MD calculations due to the relaxation of the environment, i.e., nearby groups of the W254, leading to the reduction of the energy demands. The significant lower barrier explains the experimental finding that the dihedral barrier of W254 is overcome. Furthermore, sartans were studied to evaluate their potential as furin inhibitors. Sartans are AT1 antagonists, and they effectively inhibit the hypertensive effects induced by the peptide hormone Angiotensin II. Here, they have been docked into the cavity to evaluate their effect on the BOS ligand via docking and molecular dynamics simulations. A consistent binding of sartans within the cavity during the simulation was found, suggesting that they could act as furin inhibitors. Finally, sartans interact with the same amino acids as W254, leading to a competitive binding that may influence the pharmacological efficacy and potential drug interactions of sartans.

## 1. Introduction

Furin, primarily located on the Golgi membrane, is a calcium-dependent serine endopeptidase belonging to the extensive subtilisin-like *proprotein convertase family* [1,2,3]. It facilitates the alteration and activation of several essential protein precursors (referred to as proprotein substrates), which include pathogens, growth factors, receptors, and extracellular matrix proteins [4]. Furin operates by hydrolytically cleaving oligopeptides and protein substrates at partially conserved paired recognition sequences, such as -Lys-Arg- or -Arg-Arg-. Widely located in human and other mammalian tissues, furins are crucial in many disease processes, such as diabetes [5], obesity [6], atherosclerosis [7], cancer [8], Alzheimer’s disease [9,10], and viral infections [4] such as COVID-19 [11,12,13,14]. They enhance the expression of virulence factors and promote both the pathogenesis and infectivity of various viruses and bacteria, such as Herpes viruses, Coronaviruses, Retroviruses, Diphtheria toxin, Anthrax toxin, Shigella toxins, and Pseudomonas exotoxins [2]. Furin activates the SARS-CoV-2 spike glycoprotein at specific multi-basic recognition sequences, particularly at the main S1/S2 cleavage site and at the S2’ site, with the help of the trypsin-like protease TMPRSS2 [14,15,16,17,18,19,20,21,22]. Cleavage of viral glycoproteins at these sites occurs between specific arginine-rich residues. Hence, compounds such as “arginine blockers”, which have anionic tetrazole and/or carboxyl functional groups, especially bisartans with increased acidity, hold promise as repurposed antiviral drugs [23]. Sartans, including telmisartan, candesartan, and losartan, have shown beneficial effects in COVID-19 patients with hypertension. They hinder furin activity and subsequent infection by obstructing basic amino acids at cleavage sites [24,25,26]. Furins are key targets for developing new antiviral therapies because of their role in the initial stages of cellular infection. However, targeting furins is challenging since this family of proteases is also essential to many beneficial metabolic processes. Thus, drugs designed to inhibit furin activity must selectively target furin variants involved in the disease while minimizing adverse effects on beneficial biochemical pathways [27,28,29]. 

**Figure 1:**
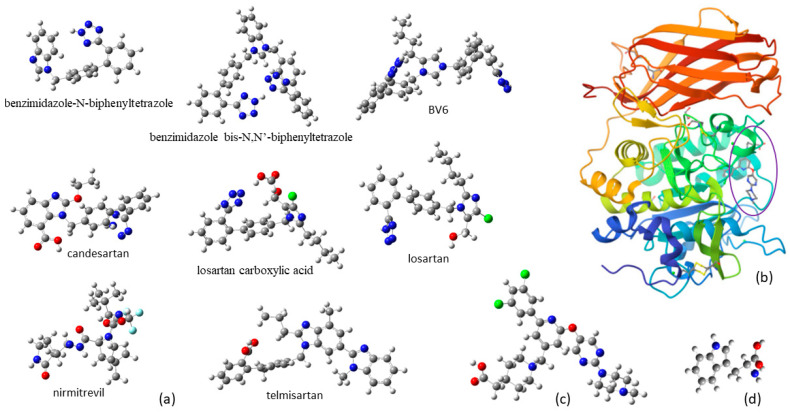
Chemical structures of (**a**) sartans, (**b**) furin enzyme, (**c**) BOS-318 ligand, and (**d**) tryptophan amino acid. (C atoms: grey balls, H: white, O: red, N: blue, F: cyan, Cl: green).

Sartans, a representative class of drugs among AT1 antagonists, selectively target the AT1 receptor, effectively inhibiting the hypertensive effects of the peptide hormone Angiotensin II (Figure 1a). Commonly administered as antihypertensive medications in the form of a water-soluble potassium salt formulation (LSR), sartans have recently been suggested as candidates for COVID-19 treatment. However, their high lipophilicity and suboptimal bioavailability require continuous efforts to enhance their formulation and leverage advancements in nanotechnology [30,31,32,33].

In this study, we describe the calculated bonding and binding energy of the “BOS” drug within the active site of furin. New experimental evidence suggests that dichlorophenyl (DCP)-pyridine “BOS” drugs can effectively inhibit human furin through an induced-fit mechanism [34]. In this mechanism, tryptophan W254 in the furin catalytic cleft (FCC) acts as a molecular gate, rotating nearly 180 degrees through a steep energy barrier around its chi-1 dihedral to an “open” orientation, revealing a hidden hydrophobic pocket. In addition to “BOS” drugs, which are known as human furin inhibitors, sartans are also studied to evaluate their potential as furin inhibitors. Sartans are AT1 antagonists and effectively inhibit the hypertensive effects induced by the peptide hormone Angiotensin II. Here, they are docked into the cavity to evaluate their effect on the BOS ligand. Thus, the main goals of this study are to evaluate and compare recent molecular mechanics calculations with current quantum mechanics calculations and to assess the effect of docked sartans on the BOS ligand [34]. 

## 2. Materials and Methods

### 2.1. Quantum Mechanics

Density functional theory (DFT) is a popularly used methodology for the study of a wide range of phenomena in a variety of systems, and nowadays DFT is one of the most used electronic-structure methodologies [35]. In some cases, DFT can approach the accuracy of high-level ab initio electronic-structure theories at a relatively low computational cost [36,37,38], while for large molecules, having more than 100 atoms has up to now been the only choice. Its success depends on the improvement and refinement of the used functionals [35], while efforts are being made for the generation of universally applicable functionals. Up to now, many functionals that are suitable for ground state properties have been developed by optimizing functionals with a simultaneously good performance for all elements, including both binding energies and barrier height [39,40]. The most used functionals are the B3LYP [41,42] and the M0X functional families [43]. In general, they describe the binding mechanism very well; however, for more tricky applications, there is a plethora of other suitable functionals. Finally, DFT predicts UV-vis absorption and fluorescent spectra [44], IR and Raman spectra [45] as well as the chemical shifts for the ^1^H NMR well, while the NMR shifts of the non-hydrogen atoms are predicted well via the DFT methodology when appropriate basis sets are used [46].

Here, the active center of furin was studied via DFT calculations and multiscaling calculations. Specifically, the W254 and W291 amino acids of furin that play an initial role in the binding mode of BOS to furin were calculated via quantum mechanics calculations, i.e., via Density Functional Theory and via multiscaling ONIOM methodology [47]. Specifically, the active center of furin with the BOS ligand including 360 atoms was geometry-optimized using the ONIOM(M06-2X/6-311+G(d,p):PM6) methodology. In the ONIOM methodology, the calculated system is defined as two regions, where the W amino acids were calculated via density functional methodology, M06-2X/6-311+G(d,p) [43,48], while the remaining active site was calculated using the semi-empirical PM6 methodology [49]. Then, for the optimized geometries, the interaction of the W254 with the 7LCU part was calculated with the M06-2X/6-311+G(d,p) method. The interactions of both the “open” and “closed” W254 residue with the active site and the energy barrier were obtained. The conformation analysis has been carried out with respect to the electronic energy. Furthermore, the reaction enthalpies ΔH have been calculated at 298.15 K and 1 atm. Specifically, the thermal corrections to enthalpy have been calculated at the PM6 level of theory, and the values have been added to the binding electronic energies. Vibrational analysis has been carried out at PM6 and ONIOM for the complexes and at the M06-2X/6-311+G(d,p) method for the W amino acid. The presently used methodologies have been successfully used in encapsulation systems [50]. All calculations were carried out in water solvent using the polarizable continuum model (PCM) [51]. The calculations and the visualization were conducted using the Gaussian 16 code [52].

### 2.2. Induced Fit Docking

Induced fit docking was utilized to explore potential binding interactions between the known AT1 antagonists and the active site of furin. The crystal structure that was used for the computational studies was PDB ID 7LCU [53]. In silico computations were carried out using the protein preparation wizard module in Schrödinger Suites to prepare the crystal structure. This module breaks existing bonds into metals and forms new zero-order bonds between metals and nearby atoms, while adjusting formal charges to match the X-ray-determined coordination geometry. Sartans were modeled using Schrödinger’s Maestro molecular modeling platform and then minimized using Macromodel54 and DFT calculations. LigPrep was utilized to prepare 3D models, taking stereochemistry into account, and the “add metal binding states” option was selected to ensure effective binding [54]. The optimization of geometries with MacroModel retained proper chiralities. The OPLS2005 [55] force field was used for minimization and the consideration of protonation states at physiological pH. Chemically reasonable 3D models were created using Hammett and Taft methodologies alongside an ionization tool. Ligand structures underwent further minimization with water as the solvent and OPLS2005 as the force field. A combination of torsional and low-sampling conformational searches was conducted to produce energetically favorable conformations, with the most optimal conformation selected for docking calculations. The induced fit docking (IFD) method in Schrödinger Suites was utilized for docking calculations. The ligand was docked into the five energetically favored conformations generated by Macromodel. Before docking, the protein preparation included constrained refinement, which involved the automatic trimming of side chains based on B-factor and Prime refinement of protein side chains. Glide/XP was used for docking, with the dielectric constant of the active site set to 80, and waters from the crystallographic data were retained during the docking process [56].

### 2.3. Molecular Dynamics

The MD studies [57] were conducted with SPC/E-modeled water molecules surrounding the drug–protein complex. Neutralization was achieved with Na^+^ and Cl^-^ ions until reaching an experimental salt concentration of 0.150 M NaCl. The protein’s N-terminus was capped with an acetyl group, while the C-terminus remained uncapped, as it is part of the protein’s active site. Protein–ligand interactions were modeled using the OPLS2005 force field, and long-range electrostatics were handled with the particle mesh Ewald method (PME) [58,59] and a grid spacing of 0.8 Å. Van der Waals and short-range electrostatic interactions were smoothly truncated at a distance of 9.0 Å. Temperature was maintained using the Nose–Hoover thermostat [60], and pressure was controlled using the Martyna–Tobias–Klein [59] method. Periodic boundary conditions were applied, and the simulation box dimensions were (10.0 × 10.0 × 10.0) Å^3^. The equations of motion were integrated by employing the multistep RESPA integrator with an inner time step of 2 fs for bonded interactions and non-bonded interactions within a 9 Å cutoff [61]. The outer time step was 6.0 fs, and it was used for non-bonded interactions beyond this cutoff. Each system was equilibrated using the default protocol provided by Desmond. An outer time step of 6.0 fs was used for non-bonded interactions beyond the cutoff. Each system underwent equilibration using the default protocol provided by Desmond [62]. The initial relaxation of the system was carried out through Brownian dynamics simulation in the NVT ensemble at 310 K, with constraints on solute-heavy atoms. Following this, relaxation in the NPT ensemble without constraints was allowed for 1.0 ns before entering the production phase of the MD simulation, which was set to 200 ns to ensure sufficient sampling for analyzing the molecule’s binding mode within the protein cavity. The MD simulations were conducted on workstations utilizing GPU-accelerated MD simulation codes, and simulation accuracy was evaluated based on the RMSD convergence of the protein backbone Ca atoms and the ligand’s RMSD.

## 3. Results and Discussion

### 3.1. Quantum Mechanics Calculations

Recently, experimental evidence showed that “BOS” drugs can sufficiently inhibit human furin by an induced-fit mechanism in which tryptophan, W254, in the FCC acts as a molecular gate, rotating about 180 degrees through a steep energy barrier around its chi-1 dihedral to an “open” orientation, exposing a hidden hydrophobic pocket [1,34]. The molecular dynamics simulations indicated that ligand entry and binding within the FCC are likely influenced by energy fluxes resulting from the disruption and reformation of solvation shells around the ligand and protein as the drug moves from the solution phase into furin catalytic cleft [34]. 

**Figure 2:**
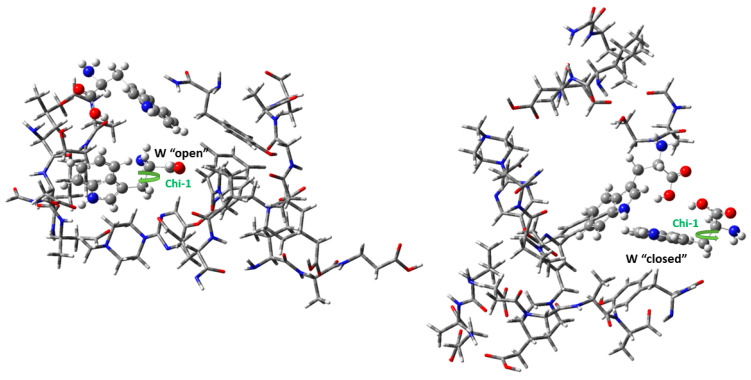
Optimized conformations of furin–BOS complex with “open” and “closed” conformation of W254 amino acids via ONIOM(M06-2X/6-311+G(d,p):PM6) method.

The conformations of “open” and “closed” tryptophan in the furin–BOS complex are calculated via DFT methodology and are shown in Figure 2. The QM calculations predict that the most stable complex is the complex that has the W254 in the “open” form. This complex is found to be 30.5 kcal/mol more stable than the complex that includes the “closed” conformation via the M06-2X/6-311+G(d,p) methodology. In the ONIOM methodology, the energy difference of the two complex conformations was smaller, i.e., 6.4 kcal/mol (Table 1). The binding energies (BEs) of the two amino acids with the furin–BOS is −32.6 kcal/mol (“open”) and −19.3 kcal/mol (“closed”), and they are calculated as E(complex)—E(furin-BOS)—E(W)—E(W). These values correspond to electronic energy. The corresponding binding enthalpies are calculated at −32.8 kcal/mol and −18.8 kcal/mol. Finally, the energy barrier for the furin–BOS complex to transit from the “closed” to the “open” structure is calculated at 30 kcal/mol at the ONIOM level of theory. This value is significantly smaller than the observed torsional barrier of ~158 kcal/mol of the MD calculations [34]; however, the QM calculations use a significantly smaller complex than the MD calculations. The small torsion barrier is attributed to the environment of the W254, where in the case of the QM calculations it can be more easily relaxed to assist the torsion barrier. Thus, QM calculations show the significant role of the relaxation of the nearby groups, which can reduce the energy demands. 

Vibrational analysis of the tryptophan amino acids does not show any imaginary vibrational mode. Their five lowest energy vibration modes range from 16 cm^−1^ to 174 cm^−1^ at the M06-2X/6-311+G(d,p) method and correspond to vibrational motions that affect the CCCC dihedral angle. In the lowest energy conformer of the free tryptophan, the H atom of the -COOH forms a hydrogen bond with the N atom of its amine group. Its five lowest energy vibrational modes range from 25 cm^−1^ to 148 cm^−1^, and in some of them, the CCCC dihedral angle is affected, less however than the vibrational motions of the W amino acids of the complex. Some of the higher energy vibrations of the complexed tryptophan also affect the CCCC dihedral angle and the transition from “closed” to “open” structure, but they are in conjunction with other motions, such as stretching of bonds, twisting, scissoring, etc. Thus, the vibrational motions of W further assist the reduction of the torsional energy requirements.

### 3.2. Induced Fit Docking

By applying the Glide/XP algorithm for induced fit docking (IFD), multiple conformations were produced for the docking of each sartan into the furin cavity. These conformations were ranked according to the IFD score, in descending order. This scoring function evaluates both the binding strength of the ligand to the protein’s active site and the Prime energy of the protein across all protein–ligand complexes. From the results, it seems that all sartans bind strongly to the active center.

**Figure 3:**
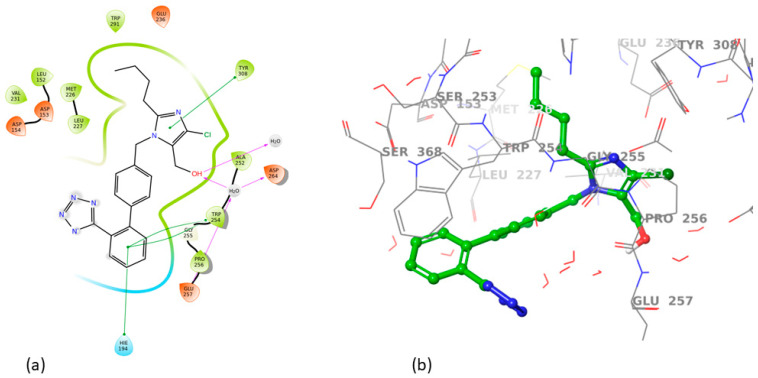
Interactions of losartan with 7LCU in (**a**) 2D and (**b**) 3D presentation.

**Figure 4:**
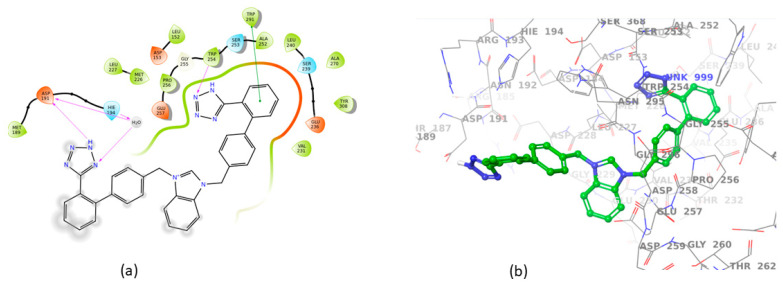
Interactions of Benzimidazole bis-N,N′-biphenyltetrazole with 7LCU in (**a**) 2D and (**b**) 3D presentation.

**Figure 5:**
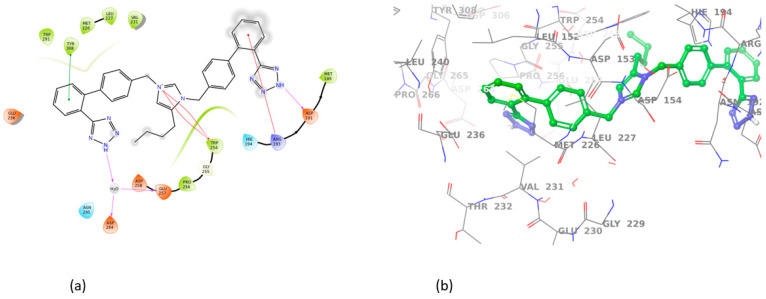
Interactions of BV6 with 7LCU in (**a**) 2D and (**b**) 3D presentation.

**Figure 6:**
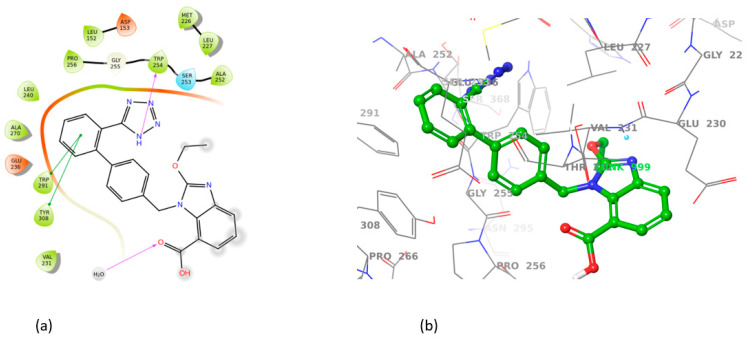
Interactions of candesartan with 7LCU in (**a**) 2D and (**b**) 3D presentation.

**Figure 7:**
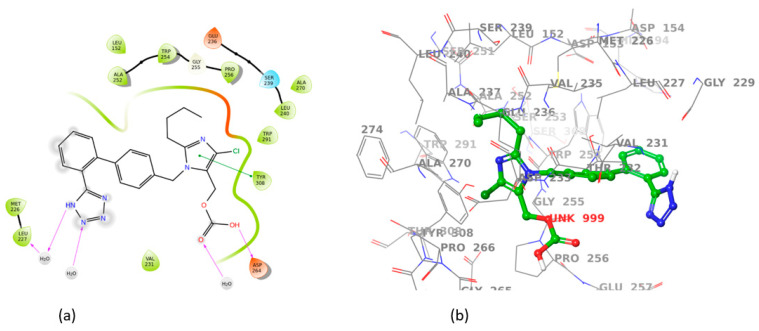
Interactions of losartan carboxylic acid with 7LCU in (**a**) 2D and (**b**) 3D presentation.

**Figure 8:**
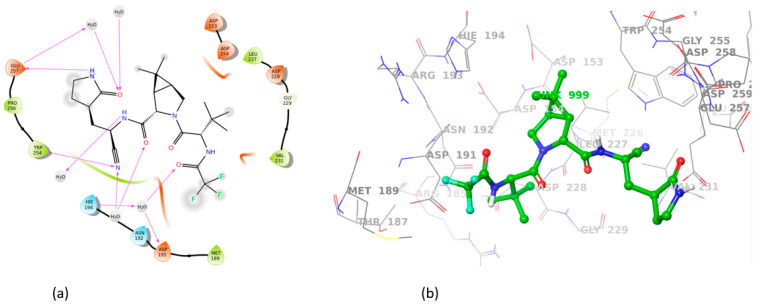
Interactions of nirmitrevil with 7LCU in (**a**) 2D and (**b**) 3D presentation.

**Figure 9:**
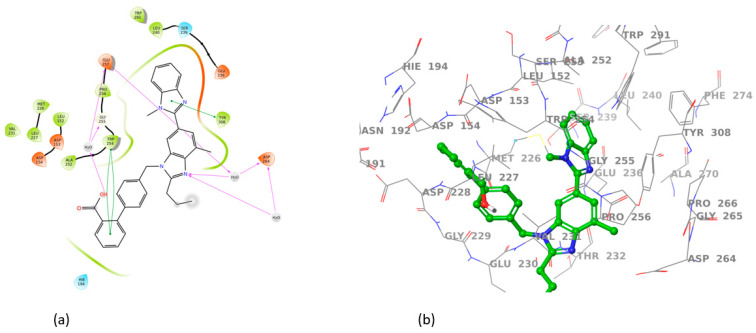
Interactions of telmisartan with 7LCU in (**a**) 2D and (**b**) 3D presentation.

**Figure 10:**
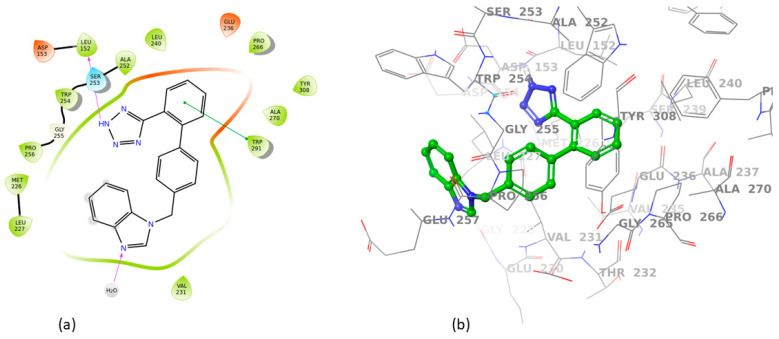
Interactions of benzimidazole-N-biphenyltetrazole with 7LCU in (**a**) 2D and (**b**) 3D presentation.

All sartans bind strongly to the cavity of furin, but the strongest affinity is shown by the benzimidazole bis-N,N′-biphenyltetrazole. Specifically, losartan forms three hydrogen bonds with alanine252, aspartic acid264, and two π-π interactions with tryptophan254 and histidine194. Moreover, Benzimidazole bis-N,N′-biphenyltetrazole forms three H-bonds with aspartic acid191, tryptophan254 and histidine194 and one π-π interaction with tryptophan291. Also, BV6 forms two hydrogen bonds with aspartic acid191 and glutamic acid 257 and two π-π interactions with arginine193 and tryptophan254. Furthermore, candesartan forms one hydrogen bond with tryptophan254 and two π-π interactions with tryptophan291 and tyrosine308. On the other hand, losartan carboxylic acid forms one hydrogen bond with aspartic acid264 and one π-π interaction with tyrosine308. Nirmitrevil forms four hydrogen bonds with aspartic acid191, histidine194, tryptophan254 and glutamic acid257. Also, telmisartan forms three hydrogen bonds with aspartic acid264, glutamic acid257 and glycine255 and two π-π interactions with tryptophan254 and tyrosine308. Finally, benzimidazole-N-biphenyltetrazole forms one H-bond with leukice152 and one π-π interaction with tryptophan291. As can be seen in Figure 3, Figure 4, Figure 5, Figure 6, Figure 7, Figure 8, Figure 9 and Figure 10, sartans and BOS ligand bind competitively to the same pocket on furin protein, interacting with identical amino acid residues. Specifically, they interact with the same amino acids as Tryptophan 254. This competitive binding may influence the pharmacological efficacy and potential drug interactions of losartan. All the interactions of sartans with furin are shown in Table 2.

### 3.3. Molecular Dynamics

Molecular Dynamics (MDs) simulations were performed to evaluate the stability of losartan’s orientations as indicated by docking studies. The MD simulation results for the protein–ligand complex showed stable binding of losartan within the furin cavity. To measure this stability, the Root Mean Square Deviation (RMSD) of losartan from its initial docking position was calculated. Throughout the 200 ns simulation, losartan remained attached to the binding site of furin. The consistent binding of losartan within the cavity during the simulation supports our docking findings, suggesting that it could act as a furin inhibitor.

**Figure 11:**
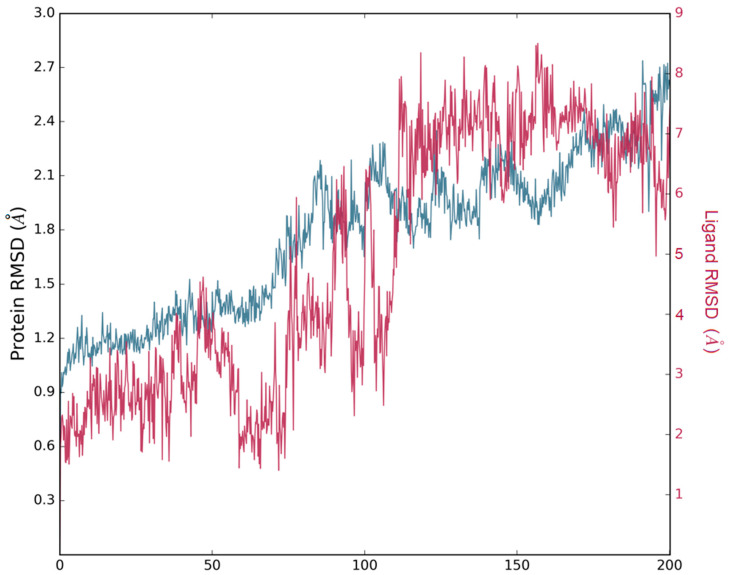
Protein’s Ca atoms display a RMSD value <8.0 Å. Blue line for the protein and red line for the ligand.

In Figure 11, the protein’s Ca atoms display a RMSD value <8.0 Å, indicating a good system convergence. The ligand’s RMSD is also shown for the 200 ns duration of the MD simulation, with the initial docking pose serving as the reference for measuring the RMSD of the ligand’s heavy atoms. Throughout the entire simulation period, the ligand maintains a remarkable stability from 120 ns to 200 ns, with an RMSD value of approximately <8.0 Å, as illustrated in Figure 11.

## 4. Conclusions

Furins are serine endoproteases that are involved in many biological processes. They play important roles in normal metabolism and in the activation of various pathogens, while they are a target for therapeutic intervention. Dichlorophenyl-pyridine “BOS” drugs can sufficiently inhibit human furin by an induced-fit mechanism. In this process, tryptophan W254 acts as a molecular transition energy gate in the furin catalytic cleft. The binding of “BOS” drug to the active center of furin has been investigated via density functional theory (DFT) and ONIOM multiscaling methodologies. The binding enthalpies (binding energies) of the W254 with the furin–BOS are −32.8(−32.6) kcal/mol (“open”) and −18.2(−19.3) kcal/mol (“closed”), while the calculated torsion barrier was found at 30 kcal/mol. It is significantly smaller than the value of previous MD calculations, i.e., ~158 kcal/mol [34]. It was proposed [34] that kinetic disturbances that affect residues near the furin catalytic cleft and entry channels, along with an enhanced kinetic energy input to the protein, could potentially lower the dihedral energy barrier of W254 and other furin residues. Therefore, this reduction can facilitate the ligand-induced rotations. Our findings here indicate that adjacent groups play a crucial role in significantly decreasing the torsional energy requirements, thereby explaining the experimental discovery that the dihedral barrier of W254 can be surpassed.

Furthermore, sartans were studied to evaluate their potential as furin inhibitors. It is known that sartans effectively inhibit the hypertensive effects induced by the peptide hormone Angiotensin II. Here, sartans were docked into the cavity to evaluate their effect on the BOS ligand via docking and molecular dynamics simulations. A consistent binding of each sartan within the cavity during the simulation was found, suggesting that they could act as furin inhibitors. Finally, they interact with the same amino acids as tryptophan 254, and this competitive binding may influence the pharmacological efficacy and potential drug interactions of sartans. Also, in a previous study, it has been shown that folic acid derivatives bind to the active center of furins [63]. Enzymatic assays and molecular docking studies have indicated that sartans, particularly those containing tetrazole groups, can bind efficiently to the ACE2–Spike RBD complex, suggesting their utility in disrupting the interaction between the virus and host cell receptors. These findings were supported by enzymatic assays showing that sartans do not inhibit ACE2 activity directly but rather block the interaction domain with the viral spike protein [29]. This comparative analysis lays the groundwork for future studies aimed at optimizing sartan-based furin inhibitors.

## Figures and Tables

**Table 1:** Dihedral angles in degrees, binding energies (BE, kcal/mol), binding enthalpies (ΔH, kcal/mol), deformation energies (Def, kcal/mol) of the complexed W254 with respect to the free W254, and relative energies (Rel, kcal/mol) of the complexes at M062X/6-311+G(d,p).

	“Open” Conformation	“Closed” Conformation
CCCC (Chi-1)	174.8	75.3
CCCC	63.3	78.9
BE	−32.6	−19.3
ΔH ^a^	−32.8	−18.8
Def	16.0	18.0
Rel	0.0	30.5

^a^ ΔH values have been calculated using the PM6 thermal correction to enthalpy.

**Table 2:** Interactions between losartan and furin cavity.

Compound	Enzyme	Hydrogen Bonds	π-π Stacking	IFD Score ^a^
Losartan	7LCU	ALA252, ASP264	TRP254, HIE194	−8.98
Benzimidazole-N-biphenyltetrazole	7LCU	LEU152	TRP291	−10.48
Benzimidazole bis-N,N′-biphenyltetrazole	7LCU	ASP191, TRP254, HIE194	TRP291	−12.06
BV6	7LCU	ASP191, GLU257	ARG193, TRP254	−10.02
Candesartan	7LCU	TRP254	TRP291, TYR308	−9.38
Losartan carboxylic acid	7LCU	ASP264	TYR308	−9.41
Nirmitrevil	7LCU	ASP191, HIE194, TRP254, GLU257		−7.30
Telmisartan	7LCU	ASP264, GLU257, GLY255	TRP254, TYR308	−11.62

^a^ In kcal/mol.

## Data Availability

Data are provided in the paper.
